# Plasma Zinc Levels in Males with Androgenetic Alopecia as Possible Predictors of the Subsequent Conservative Therapy’s Effectiveness

**DOI:** 10.3390/diagnostics10050336

**Published:** 2020-05-24

**Authors:** Irina N. Kondrakhina, Dmitry A. Verbenko, Alexander M. Zatevalov, Eugenia R. Gatiatulina, Alexandr A. Nikonorov, Dmitrij G. Deryabin, Alexey A. Kubanov

**Affiliations:** 1 State Research Center of Dermatovenereology and Cosmetology, Korolenko St., 3, bldg 6, 107076 Moscow, Russia; kondrakhina77@gmail.com (I.N.K.); verbenko@gmail.com (D.A.V.); dgderyabin@yandex.ru (D.G.D.); kubanov@list.ru (A.A.K.); 2 G.N. Gabrichevsky Research Institute for Epidemiology and Microbiology, Admiral Makarov Sr., 10, 125212 Moscow, Russia; zatevalov@mail.ru; 3 All-Russian Research Institute of Medicinal and Aromatic Plants (VILAR), Grina St., 7, 117216 Moscow, Russia; gatiatulinaer@gmail.com

**Keywords:** androgenic alopecia, zinc, minoxidil, trace elements, hormones, hair follicles

## Abstract

Androgenic alopecia (AGA) is the most common type of progressive hair loss in man. The search for reliable predictors of the conservative treatment’s effectiveness is an urgent problem today. Forty-eight patients with AGA, stages I–IV by the Norwood–Hamilton scale, were treated for 4 months with 5% topical minoxidil joints with corrections for trace element and vitamin imbalances. In most cases, the positive therapy’s effect was shown in the parietal but not in the occipital area, whereas that effect was observed in others. The attempts to associate the therapy’s effectiveness with initially defined genetic, hormonal, and metabolic parameters showed the absence of differences between groups with positive and negative outcomes. Among the studied nutrient parameters (Zn, Cu, Mg, Ca, Fe, and Se, as well as vitamins B12, E, D, and folic acid), differences between these groups was shown in zinc content only. The starting point from a zinc plasma level above 10 µmol/L likely provides the success of the subsequent conservative therapy and correlates with an increase in the hair density and diameter in the parietal area. The integral predictive value of the Zn plasma level was assessed as 72.3% (positive predictive value: −88%; and negative predictive value: −55%).

## 1. Introduction

Androgenic alopecia (AGA), also known as male pattern baldness, is the most common type of progressive hair loss. According to published data, up to 30% of men with Caucasian origin are going to get AGA by the age of 30 years, up to 50% by 50 years, and 80% by 70 years [1].

It is well known that AGA is found in genetically predisposed people, as shown in our [2] and other studies [3,4,5]. An important role in the emergence and development of this disease is also traditionally attributed to hormonal changes, mainly to an increased levels of male sex hormones [6], changing the transcription profiles in the hair follicle cells. In addition, multiple metabolic, trace element, and vitamin changes that disturb the nutrition of the hair follicle and lead to hair loss have also been discussed [7,8,9,10].

A comparison of the role of genetic and non-genetic factors shows that patients with a low level of genetic risk of AGA developing have a higher number of significant non-genetic factors, including an increased level of dihydrotestosterone, 17-OH-progesterone, and insulin, as well as deficiencies in Mg, Cu, Zn, and Se, as well as vitamins D, E, and folic acid, compared to patients with a high level of genetic risk [10]. The obtained result indicates the variability of pathogenetic pathways leading to pathological hair loss. The currently proposed methods for treatment of alopecia are based on the use of surgical techniques [11,12], which are required in the late stages of this condition, as well as a variety of conservative therapeutic approaches aimed at correcting hormonal and nutrient changes in patients with early stages of AGA [13,14,15]. The positive experience of topical minoxidil therapy, a piperidino–pyrimidine derivative (2,4-diamino-6-piperidino–pyrimidine-3-oxide) [16] that is an ATP-sensitive potassium channel agonist (K(ATP)) channels), is known [17]. Partial restoration of hair growth is indicated after the systematic use of trace elements [18,19], vitamins [20], and their combinations [15,21,22]. At the same time, the substantial price and labor costs for such treatment are not always justified, which determines the relevance of the search for early informative predictors of the effectiveness of conservative therapy.

The aim of this study was to analyze the effectiveness of conservative treatment in patients with early stages of AGA, and to compare outcomes with the initially identified genetic and non-genetic factors with the previously proven value in the occurrence and development of AGA. The obtained results should determine the list of informative predictors of a positive or negative response to such therapy.

## 2. Materials and Methods

### 2.1. Patients

This is a case-control study, in which we recruited a total of 48 males aged 18 to 55 (26.2 ± 5.3) years with early-stage AGA (stages I–IV according to the Norwood–Hamilton scale), with subsequent division into two groups depending on the outcome of the conservative treatment. The duration of the disease ranged from several months to 6 years, with an average duration of 3.2 ± 1.1 years. Criteria for non-inclusion were other forms of alopecia, as well as cases of hair loss as a complication of another (main) disease. All individuals included in the study provided written informed consent to participate in the study.The study was carried out in accordance with the rules of the Helsinki Declaration of 1975 [23], revised in 2013.The protocol was approved by the Local Ethics Committee of the State Research Center of Dermatovenereology and Cosmetology (protocol no. 7 from 10/31/2017), according to which it meets the standards of good clinical practice and evidence-based medicine.

### 2.2. Trichograms Analysis

The quantitative characteristics of the hair before and after treatment were evaluated based on trichogram and phototrichogram data using an Aramo SG micro camera (Aram HUVIS Co. Ltd., Republic of Korea), followed by processing of the obtained images with the professional diagnostic software Trichoscience PRO (version 1.4., TRILOGIC, Russia) Using a 60× lens in areas of 0.1 ± 0.004 cm^2^, the amount of hair was determined in the androgen-dependent (parietal) and androgen-independent (occipital) zones, as was the percentage ratio of terminal and vellus-like hair. Hair diameter was measured using a 200× lens. Before the procedure, hair was cut to a length of 0.2–0.3 mm in areas of 8–10 mm^2^ in the parietal and occipital regions, and in 48 hours a black IgoraBonacrom colorant (Schwartzkopf, Germany) was applied. After a 10min exposure, the dye was washed off with an alcohol-containing agent, and the stained areas were analyzed using a 60× lens. А hair shaft less than 40 microns in length was defined as telogen hair, and more than 40 microns as anagen hair. The amount of hair per square centimeters was calculated automatically.

The effectiveness of treatment was evaluated by changing the total amount of hair in the parietal and occipital areas, hair diameter, and the anagen/telogen ratio in the studied areas compared to similar parameters determined during the initial examination.

### 2.3. Conservative Treatment algorithm for AGA

All patients received 5% topical minoxidil solution two times daily. Correction of the trace element and vitamin imbalance revealed during the initial examination in patients with AGA was carried out using available pharmacological forms containing 124 mg zinc sulfate (one tablet twicedaily after meals for 2 months), 400 mg copper chelate (one tablet once daily after meals for 2 months), 50 µg selenium (one tablet twice daily after meals for 2 months), 357 mg iron (III)-hydroxide polymaltose complex (one tablet once daily after meals for 2 months), 500 mg magnesium orotate dihydrate (one tablet twice daily after meals for 2 months), 5000 IU cholecalciferol (vitamin D3) (once daily for 2 months), 5 mg folic acid (one tablet once daily after meals for 2 months), 400 mg vitamin E (once daily after meals for 2 months), and 2.0 mL Milgamma® (vitamin B complex) (10 times), as intramuscular injections every other day. The total duration of treatment was 4 months.

### 2.4. Studying of the Genetic and Non-Genetic Factors Considered as Possible Predictors of the Effectiveness of Conservative Treatment

Prior to treatment, in all patients a combination of genetic and non-genetic factors listed below was estimated. Some of non-genetic factors, corrected during therapy, were determined after its completion.

The DNA samples were extracted from whole blood and used in a polymerase chain multiplex reaction for rs5919324, rs1998076, rs929626, rs12565727, and rs756853 SNP genotyping by SNaPshot, using a single-base extension (SBE) approach with a SNaPshot Multiplex Kit (Life Technologies, Applied Biosystems, Waltham, MA, United States) as described [10].

Total and free testosterone, dihydrotestosterone, 17-OH-progesterone, androstendion, sex hormone-binding globulin, prostate-specific antigen, thyroid-stimulating hormone, and insulin determination in plasma was carried out by enzyme-linked immunosorbent assay using a MultiscanAscent microplate photometer (ThermoScientific kit, United States) with DRG Instruments GmbH reagents (Germany).

Plasma glucose (glucose oxidase method), cholesterol (cholesterol oxidase method),iron-binding protein ferritin (immunoturbidimetric method), Fe (colorimetric method with Ferene S) and Ca (with arsenazo III) concentrations were determined using a KONELAB 20XTi biochemical analyzer (ThermoScientific, United States) and commercial kits (ThermoScientific, United States) according to the manufacturer’s instructions.

Mg, Zn, Cu, Se, and trace element concentrationsin plasma were also estimated by direct colorimetric tests (ThermoScientific, United States), using a KONELAB 20XTi biochemical analyzer (ThermoScientific, United States) or atomic absorption spectrometry using AA-7000 platform (Shimadzu, Japan), according to the manufacturer’s instructions. For the determination of element concentrations, a sample preparation was performed [24], and certified reference material of human serum (Seronorm Trace Elements, Serum Level 1, 0903106, Sero AS, Norway) was used to test the accuracy of the methods.

To determine the concentrations of vitamins B12, D (in the form of 25 (OH)-D3), and E, as well asfolic acid, an enzyme-linked immunosorbent assay (EUROIMMUN AG, Germany)and immunoluminescent analysis, as well as high-performance liquid chromatography mass spectrometry using EVOQ TQ MS (Bruker Daltonics GmbH, Germany), were used.

### 2.5. Statistical Analysis

The data obtained were processed using STATISTICA 13.0 (StatSoft Inc., United States) [25]. To analyze the genetic factors of sensitivity to conservative therapy, artificial neural networks based on the principle of multilayer perceptron (MLP) were used. A pairwise comparison of the results before/after treatment was evaluated by the Wilcoxon test, and a frequency analysis according to the results of laboratory tests and data from the trichograms after treatment was performed. Group-by-group comparison of data was evaluated using the Mann–Wintney test. The level of significance was set at *p*<0.05.

To evaluate the prognostic effectiveness of the identified parameters, the positive and negative predictive values were calculated, as well as the integral indicator of significance [26].

## 3. Results

### 3.1. Clinical Characteristics of AGA and an Assessment of the Effectiveness of Conservative Therapy

An initial examination of patients’ hair loss at the frontal hairline (stage I according to the Norwood–Hamilton scale), the formation of bilateral bald patches in the frontal area and hair thinning on the parietal or crown scalp (stage II), progressive hair loss in the frontal and parietal regions (stage III), and up to the complete fusion of foci of baldness in the frontal and parietal scalp (stage IV) were recorded [2]. Analysis of the trichograms showed significant hair thinning in patients with androgenetic alopecia, manifested by a decrease in the average hair diameter in the parietal area, as well as a pronounced increase in the proportion of fluffy (vellus-like) hair. Analysis of phototrichograms showed an increase in the proportion of telogen hair in the parietal area, with the opposite tendency in the number of anagen hair.

**Figure 1:**
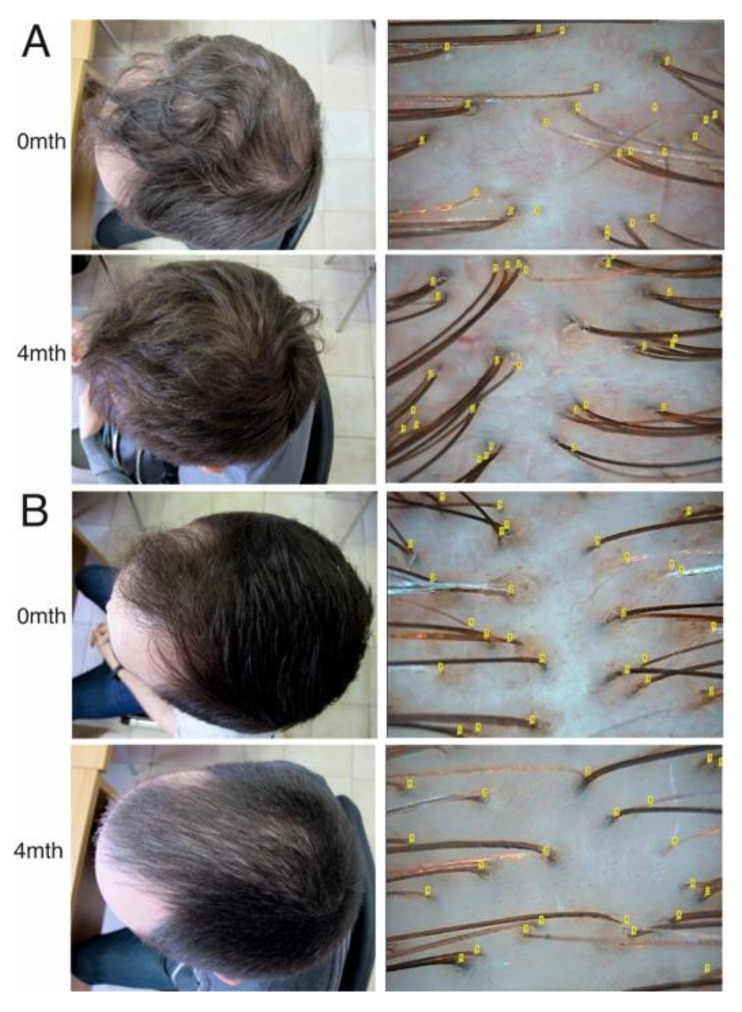
Global photos and trichograms assessment before and after 4 months of conservative treatment in androgenic alopecia (AGA) patients with a positive effect (**A**) and an absence of effect (**B**).

Typical hair loss patterns and trichograms are shown in Figure 1.

According to the results of the clinical examination, the early stages (I and II, according to the Norwood–Hamilton scale) were diagnosed in 22 patients (46%), stage III in 16 (33%), and stage IV in 10 (21%).

Four months after the completion of conservative therapy, a re-examination was performed. Trichogram analysis revealed the differences in the dynamics of the analyzed parameters in the androgen-dependent (parietal) region and androgen-independent (occipital) region in 32 patients, as a result of which the following subgroups were identified: group of patients with positive effect therapy, with improvement of the analyzed trichogram parameters (32 patients, or 67%; positive effect); and patients with an absence of effect (16 patients, or 33%), in which significant changes in the trichogram before and after treatment were not recorded (absence of effect) (Table 1). In the positive effect group, significant (*p* < 0.001) changes in the hair density and diameter, as well as an increase in the percentage of anagen hair and a decrease in telogen hair in the parietal region were observed. At the same time, there was no significant difference in hair parameters between the studied groups in the occipital region.

### 3.2. Analysis of Genetic Values in the Positive Effect and Absence of Effect Groups of Patients with AGA

A study of single-nucleotide polymorphisms (SNPs) A/G at the rs5919324, rs1998076, rs929626, rs12565727, and rs756853 loci, which are the most significant in terms of assessing the risk of AGA developing [10], showed no GG (rs12565727) genotype in patients without treatment effectiveness, (*p* = 0.002). Overall, such genotype frequency was as low as 9.4%, indicating the weak coverage of the patient cohort’s gene pool in using this single genotype as predictor for AGA classical treatment effectiveness. No differences between the groups with a positive effect and absence of effect were found for the calculated, integral-value “genetic risk” of AGA, based on combined influence of the five aforementioned SNPs. The obtained data indicate the inapplicability of the previously proposed algorithm of AGA progress and manifestation genetic risk calculation for the assessment of susceptibility to conservative treatment.

In this regard, a specific algorithm for predicting of AGA treatment effectiveness based on SNP A/G at the rs5919324, rs1998076, rs929626, rs12565727, and rs756853 loci by constructing various variants of artificial neural networks with the selection of the most productive ones was searched. An MLP (multilayer perceptron) 14-7-2 classification model showed the best performance (receiver-operating characteristic (ROC) area = 0.68), with 90.6% sensitivity but very low specificity (31.25%), indicating that the SNP data were uninformative for constructing a prediction model of patients’ sensitivity to conservative therapy.

### 3.3. Analysis of Non-Genetic Values in Positive Effect and Absence of Effect Groups of Patients with AGA

Taking into account the previously shown significance of non-genetic factors (hormones, vitamins, trace elements) in the formation of AGA [2], the same parameters were evaluated depending on the treatment results. As indicated in Table 2, there were no differences between the positive effect and absence of effect groups for most parameters.

Absence of and positive effect group comparison revealed a significant 25% decrease in Zn levels. At the same time, there was no difference in any other biochemical parameters analyzed.

### 3.4. Assessing the Predictive Effectiveness of Zn in Determining the Response to Conservative AGA Therapy

**Figure 2:**
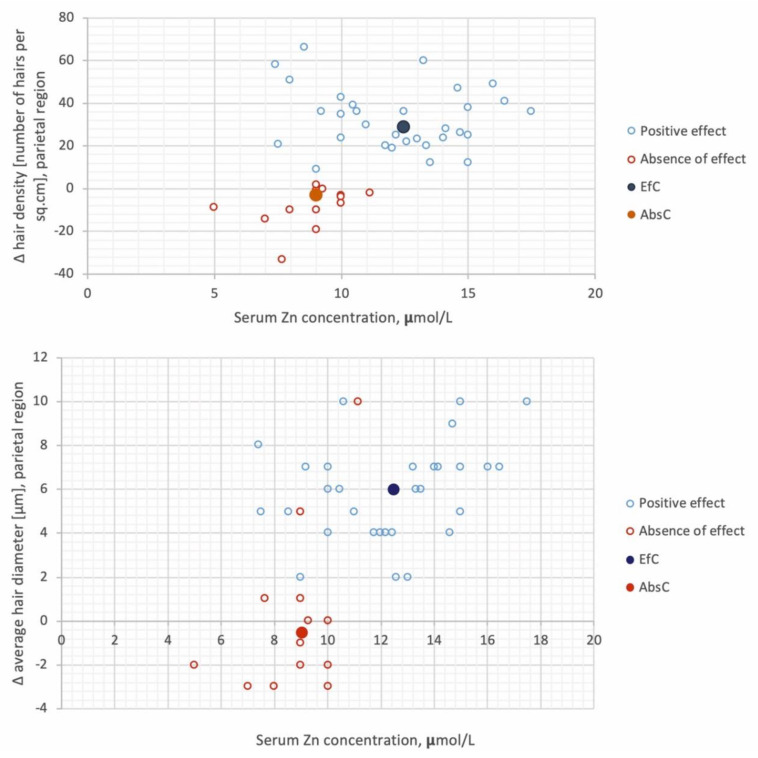
Serum zinc concentration versus change (∆) in hair density and ∆ in average hair diameter in parietal region. Medians in the studied groups are shown as EfC (positive effect) and AbsC (absence of effect).

The interrelation of the initially determined Zn level and subsequent changes in the trichogram showed the most pronounced results in the parietal region, where the Zn level was correlated with a change (∆) in hair density in all patients (general AGA cohort) with AGA (*r* (Spearman correlation coefficient) = 0.290, *p* < 0.05), as well as in the absence of effect group (*r* = 0.51, *p* < 0.05), and ∆ in average hair diameter (*r* = 0.403, *p* = 0.06) in all patients with AGA. Identified dependencies are illustrated in Figure 2.

The obtained results may indicate the possible prognostic significance of zinc levels in assessing the susceptibility of AGA patients to conservative therapy. In the case, the best separation of the studied groups depends on whether the effect of the treatment was limited, with a boundary concentration of Zn as 10 μmol/L. Using a ≤10 μmol/L threshold concentration for the absence of effect and >10 μmol/L for the positive effect, the positive and negative predictive values were 88% and 55%, respectively. Taking into account the integral calculated value of Zn plasma level significance in the prognosis of the conservative therapy mentioned above, the effectiveness was 72.3%.

### 3.5. Zn Correlation with Other Trace Elements and Vitamins

To analyze the possible mechanisms of the Zn plasma level influence on hair regrowth, a correlation analysis of the relationship between the initial level of trace elements and vitamins in the groups and their dynamics after treatment was carried out.

A negative correlation was found between the initial level of zinc and ∆ in selenium (*r* = −0.762, *p* < 0.05) in the absence of effect group and in the general AGA cohort (*r* = −0.436, *p* < 0.05), while there was no significant correlation between the initial level of trace elements and their dynamics in the positive effect group.

An analysis of the relationship between the initial Zn plasma level and the dynamics of trace elements and vitamins revealed that in the general AGA cohort, the positive effect group, and absence of effect groups, the initial Zn plasma level in individuals who had a need for Se plasma level correction was significantly lower than the control level (14.0 (12.0–15.0) μmol/L), and was 9.2 (9.0–10.0) (↓ 34%), 9.6 (9.0–13.0) (↓ 31%), and 9.0 (9.0–10.0) (↓ 36%) μmol/L, respectively. Therefore, lower Zn plasma levels in individuals with AGA may indicate a possible combined Se deficiency. The initially high Zn content in the plasma of individuals with AGA was accompanied by a more pronounced increase in vitamin E (*r* = −0.299). Intriguingly, after 4 months of conservative therapy and the correction of the decreased serum Zn levels in patients with AGA its levels did not differ significantly between the positive effect and absence of effect groups (11.56 and 11.34 μmol/L, respectively).

## 4. Discussion

The results of the study showed a significant role of zinc as a possible indicator of the effectiveness of conservative AGA therapy in males. Zinc is a structural component of more than 1000 zinc-associated transcription factors, including DNA-binding zinc finger proteins, and is required in more than 300 zinc-containing metalloenzymes [27].

It has long been suggested that Zn plays an important role in hair loss [7]. A number of studies have shown reduced serum Zn in individuals with AGA [28,29]. Rahman and Akhter showed that lower serum levels of zinc (75.41 ± 9.47 vs 99.97 ± 7.72 µg/dl in the controls) and copper (74.55 ± 9.65 vs 100.23 ± 10.95 µg/dl in the controls) may be associated with alopecia regardless of gender, and an assessment of serum Zn and Cu concentrations may be useful for the correct treatment of alopecia [30].

Chronic telogen effluvium (TE) was also associated with low levels of serum zinc [31]. On the other hand, Ozturk et al.’s study did not show a reduced serum Zn content in men with AGA, but revealed a significant decrease in hair Zn [32]. It should be noted that the exact relationship between Zn and AGA has not yet been identified, but a number of cases show that hair loss can be successfully treated with zinc supplements [18]. Progression of AGA can be effectively reduced by regular intake of L-Carnitine and zinc with dietary supplements, and topically as part of a lotion [15]. At the same time, the concentration dependence of zinc on the proliferation of the dermal papilla cells of a human follicle and the expression of regulatory proteins of the cell cycle have also been demonstrated. Tsai et al. reported that zinc chloride at a dose of 200–500 nmol significantly increases the proliferation of human follicle dermal papilla cells and the expression of cell cycle regulatory proteins [33]. However, the production of proteins associated with the cell cycle has decreased when the cells were treated with 1000 nmol zinc chloride. An increase in the apoptotic Bax protein and caspase-3 activation were observed. The results of this study suggest that zinc is involved in the regulation of proliferation or death of human dermal papilla cells in the follicle, but in a narrow dose range [33]. The fact that Zn plasma levels in the positive effect and absence of effect groups were equal upon completion of conservative treatment most likely indicates the prognostic significance of the initial Zn plasma level in relation to the effectiveness of conservative therapy, but not a decisive role in realizing the positive effect of treatment.

The results of the study expand an understanding of the role of Zn trace elements in AGA, and demonstrate their importance not only for the development of this disease, but also its sensitivity to conservative therapy. Our results show that the Zn concentration can be used as a criterion for predicting the effectiveness of conservative therapy: if the plasma concentration of Zn is ≤10 µmol/L, treatment is most likely to be ineffective; if the concentration of Zn is >10 µmol/L, treatment is likely to be effective. The calculated positive predictive value obtained in this study was 88%, the negative predictive value was 55%, and the integral predictive value was 72.3%, which confirm our recommendations on the use of Zn plasma levels as a predictor of the conservative therapy outcome. Taking this into account, the estimation of plasma Zn levels and their correction to a level above 10 µmol/L before applying conservative AGA therapy is suggested to be included to standard protocols of AGA treatment.

One of the possible mechanisms of the Zn influence on conservative therapy outcome is its participation in the metabolism of a number of trace elements and vitamins [34]. For example, competitive inhibition of iron absorption by zinc has been shown [35]. In another study, dietary Zn deficiency decreased plasma vitamin E levels [36]. Zn supplementation affected trace elements status in healthy rats [37]. In patients with chronic liver diseases, the association of Zn and other trace elements, vitamins, and hormones has been indicated [38]. Thus, the obtained data on the relationship between the initial level of zinc and the dynamics of selenium correspond to the available data on their role in the antioxidant defense system [39]. Selenium is necessary element for implementation of the activity of a number of enzymes, especially glutathionperoxidase, thioredoxinreductase, and iodothyroninedeiodinase. These selenoenzymes provide antioxidant cell defense, modification of redox status, and the conversion of the prohormone thyroxine (3,5,3′,5′-tetraiodothyronine) to the active hormone triiodothyronine [40]. Because many risk factors for hair loss, such as smoking, stress, or exposure to UV radiation, are associated with the damaging effects of reactive oxygen species (ROS) [41,42], an antioxidant defense system compromised by selenium deficiency is likely to exacerbate the effects of these risk factors [15]. This statement is also confirmed by the evidence that the lack of Zn promotes oxidative stress, and consequently, oxidative damage to DNA, proteins, and lipids [43].

Another regulating factor is vitamin E, which in addition to zinc is part of the body’s antioxidant defense system that prevents cells from free radical damage [44,45]. Bunk et al. reported that there is a relationship between zinc level in the diet and the content of α-tocopherol in plasma: a lower level of vitamin E (4.02 ± 1.20 μg/ml) in a zinc-deficient compared with a zinc-adequate diet (9.21 ± 0.70 μg/ml) [36]. Moreover, the combined use of zinc and vitamin E at a dosage of 120 mg/kg Zn plus 200 mg/kg of vitamin E in conditions accompanied by oxidative stress (for example, under the action of toxic silver nanoparticles) did not lead to an increase in glutathionperoxidase (GSH-Px) activity, and even reduced Toll-like receptor 2 (TLR2)-1 mRNA levels [46]. In our study, an initially high level of zinc may have contributed to the effective restoration of vitamin E levels, which may be due to the close relationship of zinc with the lipid metabolism [47] and fat-soluble vitamins.

Of course, the specific molecular mechanisms of the relationship between the initial level of Zn and the effectiveness of conservative AGA therapy require further study. However, taking into account the importance of this trace element in the almost all aspects of cell metabolism, and the revealed correlations between the initial Zn level and the dynamics of some trace elements and vitamins in AGA, multiple mechanisms which are dependent on the individual characteristics of the patients’ metabolism are proposed. A limitation of the present study is the small number study subjects participating. We think it is important to conduct a large-scale study, which may provide more detailed information.

In general, the obtained data demonstrate the following:

1. The algorithm for genetic risk calculation and the artificial neural networks based on SNP A/G data at the rs5919324, rs1998076, rs929626, rs12565727, and rs756853 loci did not show effectiveness in assessing the prognosis of patient sensitivity to conservative therapy;

2. There were no differences in baseline trichogram and biochemical parameters in patients with and without the effect of conservative AGA treatment;

3. Lower zinc levels in patients who showed no effects from AGA treatment;

4. The Zn level may be a relative criterion for predicting the effectiveness of conservative therapy: if the concentration of Zn ≤ 10 µmol/L, treatment is most likely to be ineffective; if the concentration of Zn > 10 µmol/L, treatment is likely to be effective;

5. The positive predictive values, negative predictive values, and the integral indicator of significance of Zn in relation to the ongoing conservative therapy were 88%, 55%, and 72.3%, respectively;

6. An initially higher serum Zn level determines a more pronounced increase in vitamin E level.

## Figures and Tables

**Table 1:** Trichogram parameters: baseline and after 4 months of conservative therapy in groups with and without the effect of AGA treatment.

Localization	Parameter	Before Treatment			4 Months after the Treatment		
		Positive Effect	Absence of Effect	*p*-Value	Positive Effect	Absence of Effect	*p*-Value
Androgen-dependent (parietal) region	Hair density (number of hairs per square cm)	190 (170–201)	188 (131–199.5)	0.285	220 (200–242.5)	170 (125–197)	<0.001
	Anagen hairs, %	70 (62–80)	69.5 (60–76.5)	0.115	85 (80–88)	70 (62.5–80)	<0.001
	Telogen hairs, %	30 (20–37)	30 (23.5–40)	0.544	15 (12–20)	30 (20–37.5)	<0.001
	Average hair diameter (μm)	43 (40–45)	41.5 (35–43)	0.568	48.5 (45–51)	40 (35–45)	<0.001
Androgen-independent (occipital) region	Hair density (number of hairs per square cm)	250 (204–263)	261 (217.5–306.5)	0.336	256 (230–287.5)	265(215–305)	0.614
	Anagen hairs, %	89 (81–93)	90 (84–96)	0.483	91.5 (87.5–95)	90 (85–95)	0.713
	Telogen hairs, %	11 (6–18,5)	10 (4–16)	0.229	8.5 (5–12.5)	10 (5–15)	0.721
	Average hair diameter (μm)	55 (54–58)	57 (54–58.5)	0.482	57 (55.5–59.5)	57 (55–60)	0.991

**Table 2:** Baseline serum biochemical and micronutrient parameters in patients showed a different response to conservative therapy.

Parameter	Group		*p*-Value
	Positive Effect	Absence of Effect	
Testesteron, nmpl/L	16.4 (10.95–24.95)	20.3 (12.75–36.5)	0.361
Testesteron free, pg/mL	16.76 (12.02–25.56)	16 (9.5–26.5)	0.696
Dihydrotestosterone, pg/mL	703.68 (571.87–1250.54)	850.14 (516.37–1406.70)	0.831
Sex hormone binding globulin, nmol/L	29.05 (21.0–45.0)	34.25 (23.0–47.8)	0.887
17-OH-progesterone, ng/mL	1.5 (1.02–2.0)	1.38 (1.0–2.0)	0.859
Androstendion, ng/mL	2.06 (1.06–3.11)	1.81 (0.94–3.30)	0.477
Thyroid stimulating hormon, µIU/mL	2.42 (2.0–3.0)	2 (1.7–2.7)	0.245
Insulin, µIU/mL	7.0 (3.0–12.9)	4.97 (3.0–10)	0.627
Prostate specific antigen, ng/mL	0.61 (0.33–1.0)	0.84 (0.64–1.00)	0.222
Cholesterol, mmol/L	4.35 (3.72–5.07)	4 (3.98–5.00)	0.803
Glucose, mmol/L	4.63 (4.25–5.0)	4.99 (4.0–5.0)	0.878
Ferretin, ng/mL	148.0 (70.95.0–204.0)	200 (118.5–287.0)	0.162
Zn, µmol/L	12.32 (10.0–14.4)	9.1 (8.5–10.6)	0.034 *
Cu, µmol/L	10.7 (9.7–13.2)	11.5 (9.5–17.5)	0.749
Mg, mmol/L	0.85 (0.76–0.97)	0.82 (0.75–1.00)	0.785
Ca, mmol/L	2.4 (2.3–2.5)	2.33 (2.3–2.4)	0.173
Fe, µmol/L	19.4 (15.0–27.3)	24.5 (21.0–29.0)	0.081
Se, µg/L	0.77 (0.55–1.0)	0.67 (0.51–1.00)	0.484
B_12_, pg/mL	319.5 (189.5–414.0)	275.0 (200.0–362.0)	0.74
E, µg/mL	6.7 (4.2–11.0)	4.6 (4.0–7.5)	0.255
D, ng/mL	24.15 (19.0–34.9)	20.5 (18.0–32.5)	0.286
Folic acid, ng/mL	4.9 (3.0–9.0)	3.69 (3.0–9.8)	0.545

Data expressed as median (25–75). * *p*-value is different between the studied groups, according to a Mann–Whitney U test.

## Abbreviations

AGA	Androgenic alopecia
MLP	Multilayer perceptron
SNPs	Single-nucleotide polymorphisms
TE	Trace elements
